# Bleeding complications in patients with out-of-hospital cardiac arrest treated with cangrelor and oral P2Y12 inhibitors

**DOI:** 10.1093/ehjacc/zuaf082

**Published:** 2025-06-06

**Authors:** Georg Gelbenegger, Alexandra Julia Lipa, Anselm Jorda, Robert Zilberszac, Gottfried Heinz, Thomas Staudinger, Christian Zauner, Michael Holzer, Guy Friedrich, Fabian Plank, Irene M Lang, Bernd Jilma, Jolanta M Siller-Matula

**Affiliations:** Department of Clinical Pharmacology, Medical University of Vienna, Vienna, Austria; Department of Medicine II, Division of Cardiology, Medical University of Vienna, Vienna, Austria; Department of Clinical Pharmacology, Medical University of Vienna, Vienna, Austria; Department of Medicine II, Division of Cardiology, Medical University of Vienna, Vienna, Austria; Department of Medicine II, Division of Cardiology, Medical University of Vienna, Vienna, Austria; Department of Medicine I, Medical University of Vienna, Vienna, Austria; Department of Medicine III, Division of Gastroenterology and Hepatology, Medical University of Vienna, Vienna, Austria; Department of Emergency Medicine, Medical University of Vienna, Vienna, Austria; Department of Medicine III, Division of Cardiology, Medical University of Innsbruck, Innsbruck, Austria; Department of Medicine III, Division of Cardiology, Medical University of Innsbruck, Innsbruck, Austria; Department of Medicine II, Division of Cardiology, Medical University of Vienna, Vienna, Austria; Department of Clinical Pharmacology, Medical University of Vienna, Vienna, Austria; Department of Medicine II, Division of Cardiology, Medical University of Vienna, Vienna, Austria

**Keywords:** P2Y12, Extracorporeal cardiopulmonary resuscitation, ECPR, Stent thrombosis, Percutaneous coronary intervention

## Abstract

**Aims:**

Cangrelor is used to bridge the gap of insufficient platelet inhibition in patients with out-of-hospital cardiac arrest (OHCA) undergoing percutaneous coronary intervention (PCI).

**Methods and results:**

In a retrospective chart review study, we investigated the incidence of bleeding and stent thrombosis in patients with OHCA undergoing PCI who received either cangrelor and transition to an oral P2Y_12_ inhibitor or an oral P2Y_12_ inhibitor alone. Subgroups consisted of patients treated with conventional cardiopulmonary resuscitation (CPR) and extracorporeal CPR. The primary endpoint was Bleeding Academic Research Consortium (BARC) 3–5 bleeding at 30 days. Between January 2016 and March 2025, 414 patients were included of which 267 received cangrelor and an oral P2Y_12_ inhibitor and 147 received an oral P2Y_12_ inhibitor alone. BARC 3–5 bleeding at 30 days occurred at a similar rate in the cangrelor group and the oral P2Y_12_ inhibitor group (18.4% vs. 19.0%, respectively; adjusted OR, 0.79; 95% CI, 0.45–1.39). BARC 3–5 bleeding at 6, 24 and 48 h was similar between the cangrelor group and the oral P2Y_12_ inhibitor group in patients treated with conventional and extracorporeal CPR. In patients treated with extracorporeal CPR, stent thrombosis occurred less frequently in the cangrelor group compared with the oral P2Y_12_ inhibitor group (2.1% vs. 4.5%, respectively; adjusted OR, 0.32, 95% CI, 0.03–3.14), but without reaching statistical significance.

**Conclusion:**

In patients with OHCA undergoing PCI, BARC 3–5 bleeding occurred at a similar rate in patients receiving either cangrelor and transition to an oral P2Y_12_ inhibitor or an oral P2Y_12_ inhibitor alone.

## Graphical Abstract

**Graphical Abstract:**
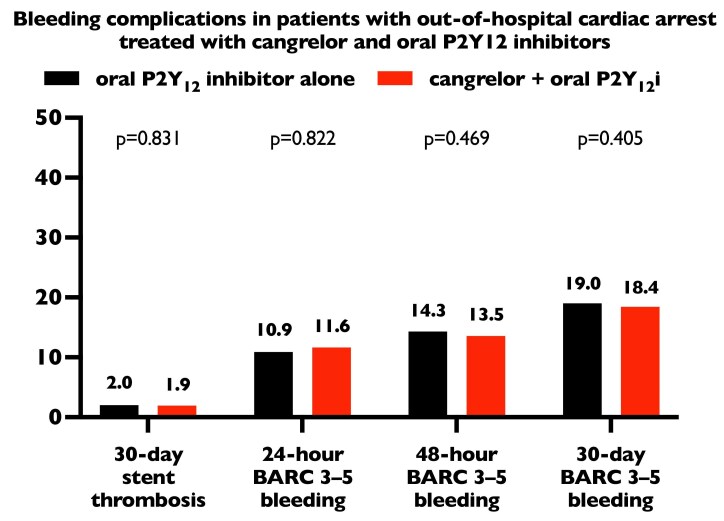

## Introduction

Acute coronary syndrome (ACS) is the leading cause of out-of-hospital cardiac arrest (OHCA).^1,2^ Immediate invasive coronary angiography and percutaneous coronary intervention (PCI) are recommended for patients with resuscitated cardiac arrest and persistent ST-segment elevation, and can be considered for patients with a suspected underlying cardiac cause.^3^ Patients with OHCA undergoing PCI face simultaneous risks of both bleeding and thrombosis.^4^ Stent thrombosis is a potentially life-threatening thrombotic complication which may occur in up to 20% of patients with OHCA.^5^ Timely and potent inhibition of platelet function in patients with OHCA undergoing PCI is critical to reduce the risk of thrombosis and improve outcome.^6^ Achieving effective platelet inhibition is challenging because of an impaired gastrointestinal absorption of oral P2Y_12_ inhibitors and hypothermia-induced changes in platelet function.^6,7^ A P2Y_12_ inhibition gap of up to 3 h was observed following administration of crushed ticagrelor via nasogastric tube in patients with OHCA undergoing PCI.^8,9^ Cangrelor is given intravenously, binds competitively to the P2Y_12_ receptor, and achieves immediate and rapidly reversible inhibition of adenosine diphosphate-induced platelet aggregation.^10^ Intravenous P2Y_12_ blockade with cangrelor in patients with OHCA undergoing PCI enabled fast and potent platelet inhibition.^11^ Pronounced platelet inhibition naturally coincides with an increased risk of bleeding which is associated with mortality, especially when it occurs early after PCI.^12^ Bleeding risk is further increased with the use of veno-arterial extracorporeal membrane oxygenation (VA-ECMO), particularly when established in cardiac arrest.^13–15^ The present study aimed to compare the occurrence rates of bleeding and stent thrombosis in patients with OHCA undergoing PCI who received either cangrelor and an oral P2Y_12_ inhibitor or an oral P2Y_12_ inhibitor alone.

## Methods

### Design

This was a retrospective, chart review study that investigated the incidence of bleeding complications and stent thrombosis in patients with OHCA undergoing PCI who received periprocedural antiplatelet treatment with either cangrelor that was transitioned to an oral P2Y_12_ inhibitor or an oral P2Y_12_ inhibitor alone between January 2016 and March 2025 at the University Hospital Vienna, Vienna, Austria (a high-volume, tertiary care centre). The study was approved by the Ethics Committee of the Medical University of Vienna (EK 1990/2023). Due to the retrospective nature, the need for informed consent was waived and data were fully anonymized prior to analysis.

### Patient population

Patients presenting with OHCA who underwent PCI and received periprocedural antiplatelet treatment with either cangrelor and an oral P2Y_12_ inhibitor or an oral P2Y_12_ inhibitor alone were retrospectively analysed. Patients were identified using the cardiac catheterization laboratory database. Data were extracted from electronic patient files and patient charts (by chart review) and entered into a database. Patients were included if they (i) presented with OHCA, (ii) underwent PCI with intracoronary stent implantation, and (iii) received periprocedural treatment with either cangrelor that was transitioned to an oral P2Y_12_ inhibitor or an oral P2Y_12_ inhibitor alone. (see Supplementary material online, *F*igure  *S1*). All patients with OHCA were treated according to advanced life support guidelines by the European Resuscitation Council.^16^ Prior to the release of the 2023 European Society of Cardiology guidelines for the management of acute coronary syndromes, no local treatment protocol dictated the use of cangrelor in patients with cardiac arrest undergoing PCI and the decision to use cangrelor was left to the treating physician. Cangrelor was given as an intravenous bolus (30 µg/kg bodyweight) followed by a continuous infusion (4 µg/kg/min). Cangrelor was transitioned to an oral P2Y_12_ inhibitor (clopidogrel, prasugrel, or ticagrelor) before, after, or at the end of cangrelor infusion. Patients treated without cangrelor received conventional periprocedural antiplatelet treatment with an oral P2Y_12_ inhibitor. Loading doses of oral P2Y_12_ inhibitors were 600 mg for clopidogrel, 60 mg for prasugrel, and 180 mg for ticagrelor. Loading doses of oral P2Y_12_ inhibitors were given as crushed/dissolved tablets via a nasogastric tube. Aspirin was administered as an intravenous bolus infusion. All patients were treated with targeted temperature management (targeted hypothermia of 33°C) for 24 h after achieving return of spontaneous circulation. A subgroup analysis was performed of patients receiving treatment with conventional CPR and extracorporeal CPR (ECPR). Patients treated with conventional CPR received manual chest compressions and/or compressions by a mechanical chest compression device. Patients treated with extracorporeal CPR received initial management with conventional CPR and femoral VA-ECMO implantation upon hospital arrival. An antegrade perfusion cannula was inserted into the superficial femoral artery ipsilateral to the arterial ECMO cannula to perfuse the distal lower extremity. Unfractionated heparin was used for anticoagulation during treatment with VA-ECMO. Patients received a bolus infusion of unfractionated heparin (5000–8000 IU) for VA-ECMO implantation, and a continuous infusion when admitted to the intensive care unit. Monitoring of anticoagulation with unfractionated heparin was performed using anti-factor Xa levels with a target level range of 0.2–0.4 IU/mL.

### Outcomes

Bleedings were classified using the Bleeding Academic Research Consortium (BARC) classification.^17^ The primary endpoint was the occurrence rate of BARC 3–5 bleeding at 30 days. To investigate bleeding occurrence during and shortly after cangrelor infusion, the occurrence of BARC 3–5 bleeding at 6, 24, and 48 h of PCI was assessed.

Secondary endpoints included stent thrombosis, cardiovascular death, bleedings according to the Thrombolysis in Myocardial Infarction (TIMI) classification (minimal, requiring medical attention, minor, and major) and BARC classification (1, 2, 3a, 3b, 3c, 4, 5a, and 5b), and bleeding site. The outcome cardiovascular death was chosen (instead of all-cause death) because all deaths that occurred in this critically ill patient population within the short-term follow-up period were attributed to a cardiovascular cause. Ischaemic outcomes including myocardial infarction, ischaemic stroke, renal artery occlusion, and peripheral artery occlusion were also recorded. The outcome stent thrombosis only included definite stent thrombosis, confirmed by coronary angiography.^18^

### Statistical methods

Continuous variables were presented as mean and standard deviation; categorical variables were reported as counts and percentages. After confirming normal distribution, between-group comparisons of continuous variables were performed using the independent *t*-test. The Fisher’s exact test was used for between-group comparisons of categorical variables. We chose to report cumulative event rates (bleeding at 6, 24, and 48 h) because determination of the exact onset of a bleeding event can be difficult using a chart review approach. Consequently, a binary logistic regression model was chosen over a Cox regression model. The binary logistic regression model was used to adjust odds ratios (OR) of the primary and secondary outcomes for age, sex, witnessed OHCA, low flow interval, and extracorporeal CPR. Unadjusted and adjusted odds ratios are shown with 95% confidence intervals (CI). We also developed univariate and multivariate logistical regression models to assess the association between baseline characteristics and the occurrence of BARC 3–5 bleeding. Candidate covariates that showed marginal associations with outcome on univariate testing (*P* ≤ 0.20), were included in the multivariate analysis for BARC 3–5 bleeding. Kaplan–Meier curves were drawn to show time-to-event analyses for BARC 3–5 bleedings. A two-tailed *P*-value of below 0.05 was considered statistically significant. Due to the exploratory nature of this study, no adjustments for multiple comparisons were performed. Statistical analyses were performed using IBM SPSS Statistics version 29 (IBM, Armonk, NY, USA) and GraphPad Prism 9.4.

## Results

This study included 414 patients, 267 received periprocedural antiplatelet treatment with cangrelor and an oral P2Y_12_ inhibitor, and 147 received an oral P2Y_12_ inhibitor alone. (see Supplementary material online, *Figure S1*) Baseline characteristics are shown in *Table 1*. Age, sex, cardiovascular risk factors, and characteristics of cardiac arrest were similar between the cangrelor group and the oral P2Y_12_ inhibitor group. ST-segment elevation was present in more than 70% of patients in each group. Treatment with extracorporeal CPR was more frequent in the cangrelor group than in the oral P2Y_12_ inhibitor group. Coronary angiography findings and antithrombotic treatments are shown in *Table 2*. Only drug-eluting stents were used. Most patients who received cangrelor were transitioned to ticagrelor (79.2%). The timing of transition from cangrelor to an oral P2Y_12_ inhibitor is shown in the Supplementary material online, *Table S1*.

**Table 1:** Baseline characteristics

	All patients	Cangrelor	Oral P2Y_12_ inhibitor	*P*-value
	*n* = 414	*n* = 267	*n* = 147	
Age (years), mean ± SD	58 ± 11	57 ± 11	59 ± 10	0.086
Male, *n* (%)	370 (89.4)	234 (87.6)	136 (92.5)	0.136
BMI (kg/m^2^), mean ± SD	28.1 ± 4.6	28.5 ± 5.0	27.6 ± 4.0	0.046
Hypertension, *n* (%)	187 (45.2)	125 (46.8)	62 (42.2)	0.409
Smoking, *n* (%)	163 (39.4)	101 (37.8)	62 (42.2)	0.402
Diabetes mellitus, *n* (%)	75 (18.1)	51 (19.1)	24 (16.3)	0.508
Dyslipidemia, *n* (%)	92 (22.2)	53 (19.9)	39 (26.5)	0.138
Family history of coronary artery disease, *n* (%)	23 (5.6)	17 (6.4)	6 (5.1)	0.378
Witnessed arrest, *n* (%)	370 (89.4)	236 (88.4)	134 (91.2)	0.410
No flow interval (min), mean ± SD	2 ± 3	2 ± 3	2 ± 3	0.106
Low flow interval (min), mean ± SD	39 ± 33	40 ± 37	37 ± 23	0.299
Intubation, *n* (%)	414 (100.0)	267 (100.0)	147 (100.0)	n/a
First rhythm shockable, *n* (%)	357 (86.4)	235 (88.3)	122 (83.0)	0.135
Epinephrine (mg), mean ± SD	4 ± 3	4 ± 3	4 ± 3	0.947
Amiodarone (mg), *n* (%)				
150	3 (0.8)	2 (0.8)	1 (0.7)	>0.999
300	104 (25.1)	67 (25.1)	37 (25.2)	>0.999
450	100 (24.2)	61 (22.9)	39 (26.5)	0.404
>450	9 (2.2)	5 (1.9)	4 (2.7)	0.727
No amiodarone	162 (39.1)	115 (43.1)	47 (32.0)	0.028
ECG changes, *n* (%)				
ECG normal	2 (0.5)	2 (0.9)	0 (0.0)	0.538
Uncertain ischaemia	113 (27.6)	75 (28.6)	38 (25.9)	0.567
ST-segment elevation	294 (71.9)	185 (70.6)	109 (74.1)	0.492
Extracorporeal CPR, *n* (%)	140 (33.8)	96 (36.0)	44 (29.9)	0.233
VA-ECMO + IMPELLA	11 (2.7)	4 (1.5)	7 (4.8)	0.059
Duration of VA-ECMO support (h), mean ± SD	92 ± 85	97 ± 94	80 ± 59	0.189

CPR, cardiopulmonary resuscitation; VA-ECMO, veno-arterial extracorporeal membrane oxygenation. Numbers and percentages do not add up to 100% because of missing data.

**Table 2:** Periprocedural characteristics and antiplatelet treatment

	All patients	Cangrelor	Oral P2Y_12_ inhibitor	*P*-value
	*n* = 414	*n* = 267	*n* = 147	
Culprit lesion, *n* (%)				
LMCA	25 (6.0)	20 (7.5)	5 (3.4)	0.130
LAD	221 (53.4)	145 (54.3)	76 (51.7)	0.681
LCX	70 (16.9)	48 (18.0)	22 (15.0)	0.494
RCA	87 (21.0)	50 (18.7)	37 (25.2)	0.132
Number of stents placed, *n* (%)				
1	254 (61.4)	166 (62.2)	88 (59.9)	0.674
2	108 (26.1)	69 (25.8)	39 (26.5)	0.907
3 or more	52 (12.6)	32 (12.0)	20 (13.6)	0.644
Thrombus aspiration, *n* (%)	37 (8.9)	19 (7.1)	18 (12.2)	0.104
Arterial access (for PCI), *n* (%)				
Transradial access	171 (41.3)	89 (33.3)	82 (55.8)	<0.001
Transbrachial access	1 (0.2)	1 (0.4)	0 (0.0)	1.000
Transfemoral access	224 (54.1)	160 (59.9)	64 (43.5)	0.001
Acetylsalicylic acid before PCI, *n* (%)	354 (85.5)	218 (81.6)	136 (92.5)	0.002
Acetylsalicylic acid during PCI, *n* (%)	52 (12.9)	40 (15.6)	12 (8.2)	0.044
Heparin before PCI, *n* (%)	352 (85.0)	218 (81.6)	134 (91.2)	0.009
Heparin during PCI, *n* (%)	347 (86.1)	219 (85.2)	128 (87.7)	0.551
Glycoprotein IIb/IIIa inhibitor before PCI, *n* (%)	0 (0.0)	0 (0.0)	0 (0.0)	n/a
Glycoprotein IIb/IIIa inhibitor during PCI, *n* (%)	23 (5.7)	7 (2.7)	16 (11.0)	0.001
Thrombolysis before PCI, *n* (%)	23 (5.6)	14 (5.2)	9 (6.1)	0.823
Cangrelor infusion interval [min], mean ± SD	n/a	146 ± 66	n/a	n/a
P2Y_12_ inhibitor following PCI, *n* (%)				
Clopidogrel	26 (6.3)	11 (4.1)	15 (10.2)	0.019
Prasugrel	52 (12.6)	29 (10.9)	23 (15.6)	0.166
Ticagrelor	328 (79.2)	222 (83.1)	106 (72.1)	0.011

PCI, percutaneous coronary intervention; LMCA, left main coronary artery; LAD, left anterior descending artery; LCX, left circumflex artery; RCA, right coronary artery.

Numbers and percentages do not add up to 100% because of missing data.

### Bleeding, stent thrombosis, and cardiovascular death

Overall, the primary outcome BARC 3–5 bleeding occurred in 77 (18.6%) of 414 patients. Eight (1.9%) patients had stent thrombosis, and cardiovascular death at 30 days occurred in 182 (44.0%) patients. (*Table 3*)

**Table 3:** Bleeding, stent thrombosis and cardiovascular death

	Overall	Cangrelor	Oral P2Y_12_ inhibitor	Odds ratio (95% CI)	Adjusted odds ratio (95% CI)^a^
	*n* = 414	*n* = 267	*n* = 147		
BARC 3–5 bleeding, *n* (%)					
At 6 h	41 (9.9)	25 (9.4)	16 (10.9)	0.85 (0.44–1.64)	0.74 (0.37–1.50)
At 24 h	47 (11.4)	31 (11.6)	16 (10.9)	1.08 (0.57–2.04)	0.92 (0.46–1.84)
At 48 h	57 (13.8)	36 (13.5)	21 (14.3)	0.94 (0.52–1.67)	0.79 (0.42–1.49)
At 30 days	77 (18.6)	49 (18.4)	28 (19.0)	0.96 (0.57–1.60)	0.79 (0.45–1.39)
Cardiovascular death, *n* (%)					
At 24 h	38 (9.2)	27 (10.1)	11 (7.5)	1.39 (0.67–2.89)	1.27 (0.59–2.75)
At 48 h	52 (12.6)	38 (14.2)	14 (9.5)	1.58 (0.82–3.02)	1.48 (0.75–2.93)
At 30 days	182 (44.0)	124 (46.4)	58 (39.5)	1.33 (0.88–2.00)	1.38 (0.85–2.24)
Stent thrombosis at 30 days, *n* (%)	8 (1.9)	5 (1.9)	3 (2.0)	0.92 (0.22–3.89)	0.85 (0.20–3.72)

BARC, Bleeding Academic Research Consortium.

^a^adjusted for age, sex, witnessed OHCA, low flow interval and extracorporeal CPR.

**Figure 1:**
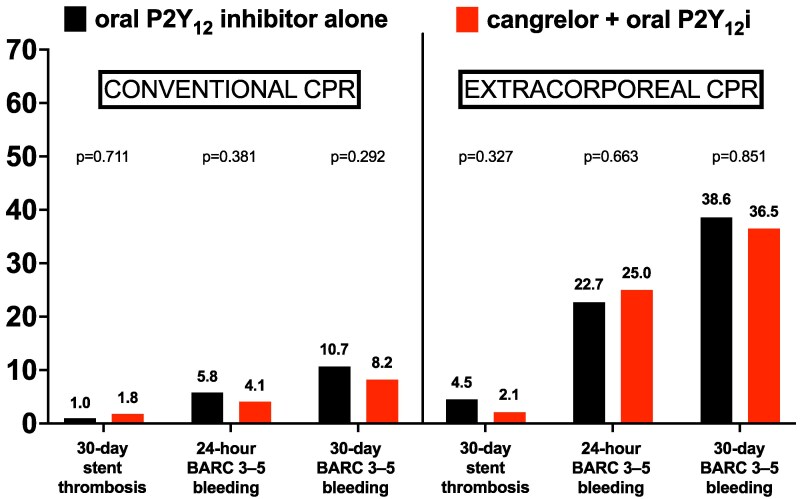
Comparison of stent thrombosis and Bleeding Academic Research Consortium 3–5 bleeding at 24 h and 30 days in patients with out-of-hospital cardiac arrest treated with either cangrelor and an oral P2Y_12_ inhibitor or an oral P2Y_12_ inhibitor alone according to treatment with conventional and extracorporeal cardiopulmonary resuscitation.

**Figure 2:**
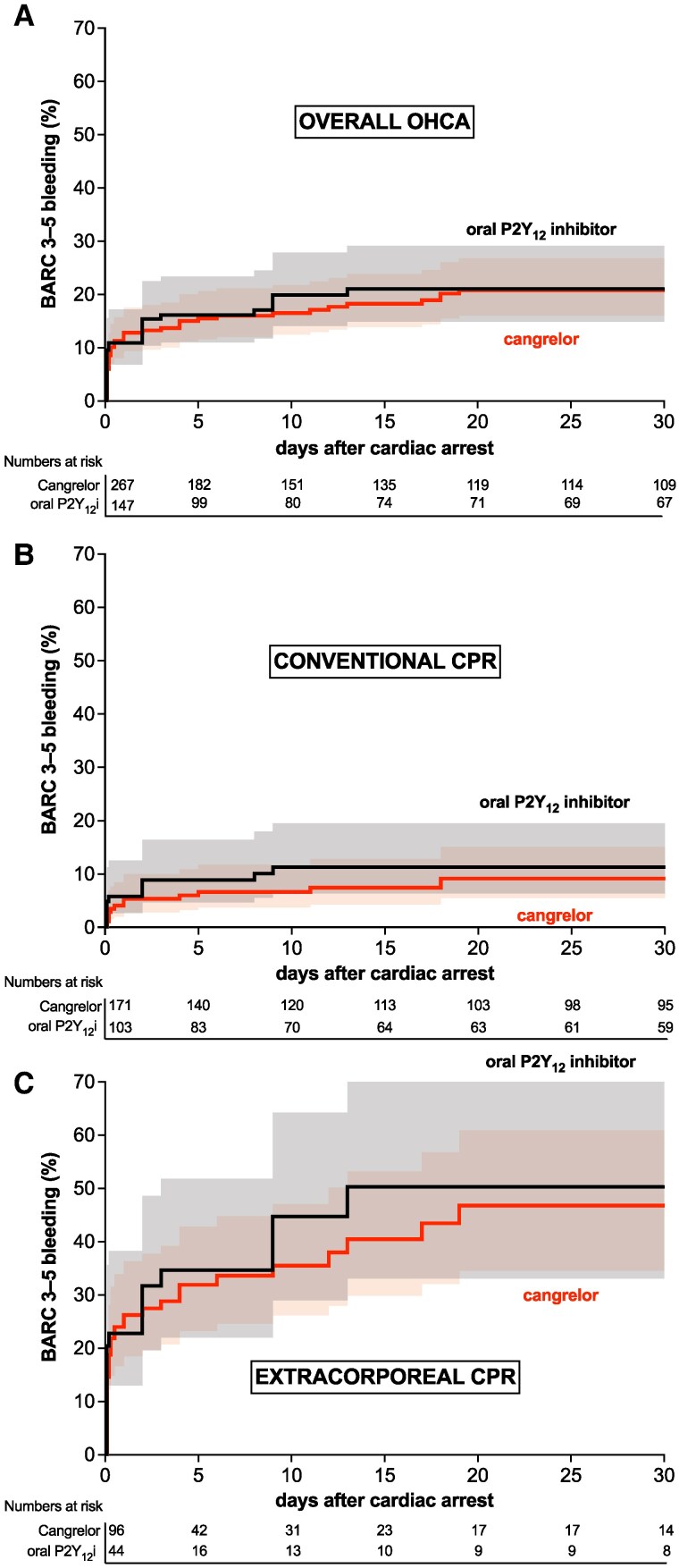
Kaplan–Meier estimates of the probability of Bleeding Academic Research Consortium 3–5 bleeding between patients with OHCA treated with cangrelor and oral P2Y_12_ inhibitors (*A*) in the overall OHCA population, (*B*) in patients treated with conventional cardiopulmonary resuscitation, and (*C*) in patients treated with extracorporeal cardiopulmonary resuscitation.

BARC 3–5 bleeding at 30 days occurred in 49 (18.4%) of 267 patients in the cangrelor group and in 28 (19.0%) of 147 patients in the oral P2Y_12_ inhibitor group (adjusted OR, 0.79, 95% CI, 0.45–1.39, *Table 3*). The incidence of BARC 3–5 bleeding at 6, 24, and 48 h was similar between the cangrelor group and the oral P2Y_12_ inhibitor group (9.4% vs. 10.9%, respectively, adjusted OR, 0.74, 95% CI, 0.37–1.50; 11.6% vs. 10.9%, respectively, adjusted OR, 0.92, 95% CI, 0.46–1.84; and 13.5% vs. 14.3%, respectively, adjusted OR, 0.79, 95% CI, 0.42–1.49). *Figure 1* compares the occurrence rates of stent thrombosis and BARC 3–5 bleeding between the cangrelor group and the oral P2Y_12_ inhibitor group, stratified by treatment with conventional and extracorporeal CPR. Kaplan–Meier curves for the primary outcome BARC 3–5 bleeding according to the treatment group are shown in *Figure 2*. Most common BARC 3–5 bleedings were cannulation and puncture site bleedings (53.2%), thorax/pulmonary bleedings (31.2%), and gastrointestinal bleedings (18.2%) (see Supplementary material online, *Figure S2*). Results of the univariate and multivariate logistic regression model are shown in the Supplementary material online, *Table S2*. After elimination of covariates, variables that were significantly associated with BARC 3–5 bleeding included a medical history of diabetes, administration of pre-PCI thrombolysis, and treatment with extracorporeal CPR. Secondary bleeding outcomes and platelet counts of patients with BARC 3–5 bleeding are shown in the Supplementary material online, *Table S3* and *Figure S3*. Patients with BARC 3–5 bleeding had a higher risk of cardiovascular death as compared with patients with a BARC 1–2 bleeding or no bleeding (see Supplementary material online, *Figure S4*). Stent thrombosis occurred in 5 (1.9%) of 267 patients in the cangrelor group and in 3 (2.0%) of 147 patients in the oral P2Y_12_ inhibitor group (adjusted OR, 0.85, 95% CI, 0.20–3.72, *Table 3*) All but one patient with stent thrombosis died at 30 days. Cardiovascular death at 30 days occurred in 124 (46.4%) of 267 patients in the cangrelor group and 58 (39.5%) of 147 patients in the oral P2Y_12_ inhibitor group (adjusted OR, 1.38, 95% CI, 0.85–2.24; *Table 3*, Supplementary material online, *Figure S5*). More ischaemic outcomes are shown in the Supplementary material online, *Table S4*.

### Conventional cardiopulmonary resuscitation

**Figure 3:**
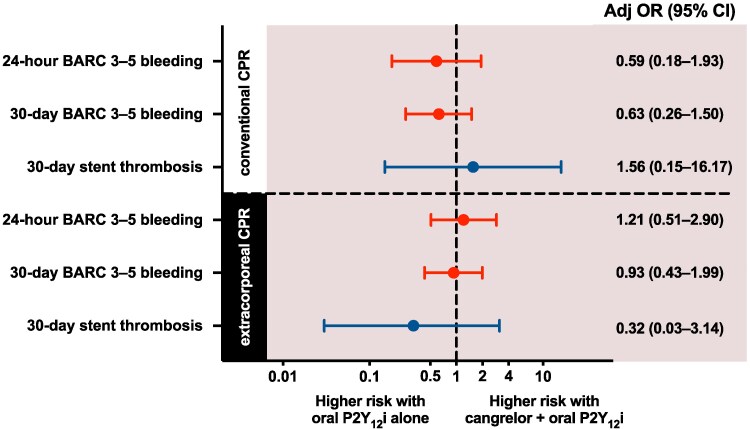
Adjusted odds ratios for Bleeding Academic Research Consortium 3–5 bleeding and stent thrombosis comparing patients with out-of-hospital cardiac arrest treated with either cangrelor and an oral P2Y_12_ inhibitor or an oral P2Y_12_ inhibitor alone, stratified by conventional cardiopulmonary resuscitation and extracorporeal cardiopulmonary resuscitation.

274 patients were treated with conventional CPR. (*Table 4*) In patients treated with conventional CPR, BARC 3–5 bleeding at 30 days occurred in 14 (8.2%) of 171 patients in the cangrelor group and in 11 (10.7%) of 103 patients in the oral P2Y_12_ inhibitor group (adjusted OR 0.63, 95% CI, 0.26–1.50; *Figure 3*). BARC 3–5 bleeding at 6, 24, and 48 h in patients treated with conventional CPR occurred at similar rates in the cangrelor group and oral P2Y_12_ inhibitor group (2.9% vs. 5.8%; 4.1% vs. 5.8%; and 5.3% vs. 8.7%, respectively). No statistically significant differences in stent thrombosis or cardiovascular death were observed between the groups.

**Table 4:** Bleeding, stent thrombosis and cardiovascular death in patients with out-of-hospital cardiac arrest according to management with conventional and extracorporeal CPR

Conventional CPR	Overall*n* = 274	Cangrelor*n* = 171	Oral P2Y_12_ inhibitor*n* = 103	Odds ratio (95% CI)	Adjusted odds ratio (95% CI)^a^
BARC 3–5 bleeding, *n* (%)					
At 6 h	11 (4.0)	5 (2.9)	6 (5.8)	0.49 (0.15–1.64)	0.49 (0.14–1.70)
At 24 h	13 (4.7)	7 (4.1)	6 (5.8)	0.69 (0.23–2.11)	0.59 (0.18–1.93)
At 48 h	18 (6.6)	9 (5.3)	9 (8.7)	0.58 (0.22–1.51)	0.50 (0.18–1.36)
At 30 days	25 (9.1)	14 (8.2)	11 (10.7)	0.75 (0.33–1.71)	0.63 (0.26–1.50)
Cardiovascular death, *n* (%)					
At 24 h	15 (5.5)	11 (6.4)	4 (3.9)	1.70 (0.53–5.50)	1.70 (0.48–6.09)
At 48 h	21 (7.7)	15 (8.8)	6 (5.8)	1.55 (0.58–4.14)	1.89 (0.61–5.86)
At 30 days	78 (28.5)	47 (27.5)	31 (30.1)	0.88 (0.51–1.51)	1.04 (0.56–1.95)
Stent thrombosis at 30 days, *n* (%)	4 (1.5)	3 (1.8)	1 (1.0)	1.82 (0.19–17.75)	1.56 (0.15–16.17)

Extracorporeal CPR	*n* = 140	*n* = 96	*n* = 44		
BARC 3–5 bleeding, *n* (%)					
At 6 h	30 (21.4)	20 (20.8)	10 (22.7)	0.90 (0.38–2.12)	0.94 (0.39–2.29)
At 24 h	34 (24.3)	24 (25.0)	10 (22.7)	1.13 (0.49–2.63)	1.21 (0.51–2.90)
At 48 h	39 (27.9)	27 (28.1)	12 (27.3)	1.04 (0.47–2.32)	1.08 (0.47–2.48)
At 30 days	52 (37.1)	35 (36.5)	17 (38.6)	0.91 (0.44–1.90)	0.93 (0.43–1.99)
Cardiovascular death, *n* (%)					
At 24 h	23 (16.4)	16 (16.7)	7 (15.9)	1.06 (0.40–2.79)	1.11 (0.41–3.05)
At 48 h	31 (22.1)	23 (24.0)	8 (18.2)	1.42 (0.58–3.48)	1.58 (0.63–3.98)
At 30 days	104 (74.3)	77 (80.2)	27 (61.4)	2.55 (1.16–5.61)	2.84 (1.23–6.52)
Stent thrombosis at 30 days, *n* (%)	4 (2.9)	2 (2.1)	2 (4.5)	0.45 (0.06–3.28)	0.32 (0.03–3.14)

BARC, Bleeding Academic Research Consortium; CPR, cardiopulmonary resuscitation.

^a^adjusted for age, sex, witnessed OHCA and low flow interval.

### Extracorporeal cardiopulmonary resuscitation

Following initial management with conventional CPR, 140 patients were treated with extracorporeal CPR. (*Table 4*) In patients treated with extracorporeal CPR, BARC 3–5 bleeding events were common and occurred in more than one-third of the patients.

In patients treated with extracorporeal CPR, BARC 3–5 bleeding occurred in 35 (36.5%) of 96 patients in the cangrelor group and in 17 (38.6%) of 44 patients in the oral P2Y_12_ inhibitor group (adjusted OR 0.93, 95% CI, 0.43–1.99; *Figure 3*). The occurrence of BARC 3–5 bleeding at 6, 24, and 48 h was comparable between the cangrelor group and the oral P2Y_12_ inhibitor group (20.8% vs. 22.7%; 25.0% vs. 22.7%; and 28.1% vs. 27.3%, respectively). In patients treated with extracorporeal CPR, stent thrombosis occurred proportionally less frequently in the cangrelor group as compared with the oral P2Y_12_ inhibitor group (2.1% vs. 4.5%, respectively; adjusted OR, 0.32, 95% CI, 0.03–3.14), however, without evidence of statistical significance. All patients treated with extracorporeal CPR who had stent thrombosis were dead at 30 days.

## Discussion

In this chart review study including a total of 414 patients with OHCA undergoing PCI, BARC 3–5 bleeding at 30 days occurred at a similar rate in patients receiving either cangrelor and an oral P2Y_12_ inhibitor or an oral P2Y_12_ inhibitor alone. Likewise, the occurrence rate of early BARC 3–5 bleeding was similar between groups, suggesting no increased risk of early bleeding with cangrelor.

Our findings agree with those of a previous study that found a comparable bleeding risk in patients with cardiogenic shock who received either cangrelor or an oral P2Y_12_ inhibitor.^19^ Results from two other retrospective studies of cangrelor in cardiogenic shock suggest that cangrelor may be safe to use in high-risk ACS patients.^20,21^ In contrast to previous literature, which only included a limited number of patients with cardiac arrest, the present study explicitly focused on patients with OHCA, who represent a different patient population than patients with cardiogenic shock who have not been resuscitated. In comparison, patients with OHCA may suffer severe traumatic injury from either the initial collapse or chest compressions, predisposing them to bleeding.^22^ Implantation of VA-ECMO during cardiac arrest, rather than during spontaneous circulation, may further increase the risk of bleeding complications.^14,23^

While the incidence of stent thrombosis in patients with chronic or even ACS (without cardiac arrest) undergoing PCI rarely exceeds 1%, its occurrence in patients with OHCA is markedly higher.^24^ Given the considerable variability in the incidence of stent thrombosis in patients with OHCA undergoing PCI,^5^ the overall incidence of stent thrombosis in the present study was within the range and there was no evidence of a statistically significant difference in stent thrombosis between the cangrelor group and the oral P2Y_12_ inhibitor group. However, patients treated with extracorporeal CPR who received cangrelor and an oral P2Y_12_ inhibitor were less likely to develop stent thrombosis as compared with patients who received an oral P2Y_12_ inhibitor alone (2.1% vs. 4.5%), but without evidence of statistical significance. In addition to a delayed onset of action of oral P2Y_12_ inhibitors, the increased risk of stent thrombosis in patients treated with extracorporeal CPR may be related to coagulopathy associated with ECMO. Coagulopathy associated with ECMO is complex, multifactorial, and quickly developing and includes platelet activation, haemostatic activation, and clotting factor consumption, which may put patients at risk for stent thrombosis.^25^ Cangrelor, used as a bridging strategy to overcome the initial P2Y_12_ inhibition gap, might counteract ECMO-associated platelet activation and help prevent thrombotic events. Cangrelor effectively bridged the gap of insufficient platelet inhibition in a randomized, controlled pharmacodynamic trial of patients with OHCA undergoing PCI, but the trial excluded patients with extracorporeal CPR and was underpowered to detect differences in clinical outcomes.^26^

Extracorporeal CPR is a rescue strategy for carefully selected patients with refractory cardiac arrest who do not respond to conventional CPR. Although patients requiring extracorporeal CPR have a low chance of survival without it, extracorporeal CPR itself imposes a considerable risk of potentially fatal bleeding and thrombotic complications.^13^ There is limited evidence on the combined effect of treatment with VA-ECMO and cangrelor on the incidence of bleeding.^27,28^ The findings of our study suggest a high occurrence rate of BARC 3–5 bleeding in patients with OHCA treated with extracorporeal CPR irrespective of the choice of antiplatelet treatment. Prospective studies of cangrelor in patients with OHCA treated with extracorporeal CPR undergoing PCI are needed to adequately investigate its effects on bleeding and thrombosis.

This study has several limitations. The results must be interpreted with caution because of the relatively small sample size and the single-centre, retrospective design.

Event adjudication was based on chart review alone, which introduces a potential for bias. Further, the results might be affected by treatment selection bias, which is inevitably introduced by the attending physicians. The more frequent use of ticagrelor in the cangrelor group and periprocedural glycoprotein IIb/IIIa inhibitors in the oral P2Y_12_ inhibitor group may have impacted the bleeding analysis. Recommendations on switching between P2Y_12_ inhibitors including transitioning from cangrelor to oral P2Y_12_ inhibitors were only published in late 2017,^29^ which might have impacted clinical practice. However, changes in clinical practice may impact both groups. Our study does not report on patient ethnicity, previous bleeding episodes, ongoing anticoagulant treatment, the total dose of periprocedural heparin administered or TIMI flow before and after PCI. The occurrence of minor bleedings may have been underestimated because they are not always mentioned in the notes. Our study was likely underpowered to detect between-group differences in the occurrence rate of stent thrombosis. Further, the rather low occurrence rate of stent thrombosis in our study might suggest potential underdiagnosing of stent thrombosis due to lack of repeat coronary angiography and autopsy with histopathological analysis.

## Conclusion

In patients with OHCA undergoing PCI, BARC 3–5 bleeding occurred at a similar rate in patients receiving either cangrelor and an oral P2Y_12_ inhibitor or an oral P2Y_12_ inhibitor alone.

## Supplementary Material

zuaf082_Supplementary_Data

## Data Availability

Additional data can be made available upon reasonable request to the corresponding author.
