# Supplementary material for: Bleeding complications in patients with out-of-hospital cardiac arrest treated with cangrelor and oral P2Y12 inhibitors
Source: Eur Heart J Acute Cardiovasc Care. 2025 Jun 6;15(4):267–75. doi: 10.1093/ehjacc/zuaf082 (PMC13123695; doi:10.1093/ehjacc/zuaf082)
Supplement: zuaf082_Supplementary_Data [file zuaf082_supplementary_data.pdf]

Supplementary material

Bleeding complications in patients with out-of-hospital cardiac arrest treated with cangrelor and oral P2Y12 inhibitors

Table of Contents

Supplementary table S1 Timing of transition from cangrelor to an oral $P2Y_{12}$ inhibitor.
Supplementary table S2. Univariate and multivariate analysis of factors associated with BARC 3–5 bleeding in patients with out-of-hospital cardiac arrest.
Supplementary table S3 Comparison of bleeding outcomes according to the TIMI and BARC bleeding definitions between patients with out-of-hospital cardiac arrest treated with cangrelor and oral $P2Y_{12}$ inhibitors.
Supplementary table S4 Comparison of ischemic outcomes between patients with out-of-hospital cardiac arrest treated with cangrelor and oral $P2Y_{12}$ inhibitors.

Supplementary figure S1 Study flowchart.
Supplementary figure S2 Bleeding sites of BARC 3–5 bleedings in the overall out-of-hospital cardiac arrest population, patients treated with conventional CPR and patients treated with extracorporeal CPR.
Supplementary figure S3 Platelet counts over the first 72 hours of admission in patients with a BARC 3–5 bleeding event.
Supplementary figure S4 Kaplan-Meier estimates of the probability of cardiovascular death in patients with out-of-hospital cardiac arrest who had a BARC 1–2 or no bleeding event versus a BARC 3–5 bleeding event.
Supplementary figure S5 Kaplan-Meier estimates of the probability of cardiovascular death in patients with out-of-hospital cardiac arrest treated with cangrelor and oral $P2Y_{12}$ inhibitors.

Supplementary table S1. Timing of transition from cangrelor to an oral $P2Y_{12}$ inhibitor.

Timing of transition	n	mean ± SD
Cangrelor to clopidogrel		
Cangrelor infusion interval [min]		158 ± 63
Before discontinuation [min]	4	87 ± 93
At discontinuation	3	
Delay [min]	3	6 ± 7
Cangrelor to prasugrel		
Cangrelor infusion interval [min]		138 ± 60
Before discontinuation [min]	17	55 ± 53
At discontinuation	5	
Delay [min]	2	26 ± 28
Cangrelor to ticagrelor		
Cangrelor infusion interval [min]		147 ± 68
Before discontinuation [min]	161	89 ± 80
At discontinuation	30	
Delay [min]	14	132 ± 176

Ticagrelor was given after discontinuation of cangrelor in 14 patients. In two of these patients, administration of ticagrelor was likely delayed intentionally because of immediate and severe bleeding. In the remaining twelve patients, the reason for delay of ticagrelor was unknown.

Stent thrombosis occurred in 8 patients. Three cases were associated with transition from cangrelor to prasugrel, two cases were associated with transition from cangrelor to ticagrelor, and three cases were associated with ticagrelor alone.

Supplementary table S2. Univariate and multivariate analysis of factors associated with BARC 3–5 bleeding in patients with out-of-hospital cardiac arrest.

**Univariate analysis**	**Odds Ratio**	**95% CI**	**p value**
Age	0.98	0.96–1.00	0.113
Sex	1.03	0.46–2.32	0.940
BMI	0.99	0.94–1.05	0.787
Diabetes	0.40	0.17–0.90	0.027
Dyslipidemia	0.74	0.39–1.39	0.346
Smoking	0.80	0.48–1.34	0.392
Hypertension	0.68	0.41–1.14	0.144
Witnessed OHCA	1.03	0.46–2.32	0.940
First rhythm shockable	0.82	0.41–1.63	0.565
No flow interval	0.95	0.86–1.04	0.281
Low flow interval	1.02	1.01–1.03	<0.001
Total epinephrine dose	1.18	1.09–1.27	<0.001
Extracorporeal CPR	5.89	3.45–10.05	<0.001
Duration of VA-ECMO support	1.00	1.00–1.01	0.052
Pre-PCI thrombolysis	5.47	2.31–12.93	<0.001
Periprocedural GP IIb/IIIa inhibitor	0.40	0.09–1.72	0.216
Transfemoral access	1.86	1.11–3.12	0.019
Cangrelor	0.96	0.57–1.60	0.862
Ticagrelor	0.91	0.50–1.66	0.754
**Multivariate analysis**	**Adj OR**	**95% CI**	**p value**
Diabetes	0.27	0.10–0.76	0.013
Low flow interval	1.01	0.99–1.02	0.268
Total epinephrine dose	0.97	0.87–1.08	0.614
Transfemoral access	1.03	0.55–1.93	0.918
Pre-PCI thrombolysis	8.24	2.79–24.30	<0.001
Extracorporeal CPR	6.70	2.89–15.53	<0.001

Supplementary table S3. Comparison of bleeding outcomes according to the TIMI and BARC bleeding definitions between patients with out-of-hospital cardiac arrest treated with cangrelor and oral $P2Y_{12}$ inhibitors.

	**OVERALL** n=414	**CANGRELOR** n=267	**ORAL $P2Y_{12}$ INHIBITOR** n=147	**Odds Ratio** (95% CI)	**Adjusted Odds Ratio** (95% CI)*
Any bleeding, n (%)	137 (33.1)	80 (30.0)	57 (38.8)	0.68 (0.44–1.03)	0.49 (0.30–0.81)
**TIMI bleeding**					
Minimal, n (%)	16 (3.9)	6 (2.2)	10 (6.8)	0.32 (0.11–0.89)	0.31 (0.11–0.90)
Requiring medical attention, n (%)	45 (10.9)	25 (9.4)	20 (13.6)	0.66 (0.35–1.23)	0.54 (0.27–1.08)
Minor, n (%)	34 (8.2)	19 (7.1)	15 (10.2)	0.67 (0.33–1.37)	0.59 (0.28–1.24)
Major, n (%)	44 (10.6)	30 (11.2)	14 (9.5)	1.20 (0.62–2.35)	0.99 (0.48–2.03)
**BARC bleeding**					
BARC 1, n (%)	15 (3.6)	5 (1.9)	10 (6.8)	0.26 (0.09–0.78)	0.26 (0.09–0.79)
BARC 2, n (%)	49 (11.8)	27 (10.1)	22 (15.0)	0.64 (0.35–1.17)	0.52 (0.27–1.01)
BARC 3a, n (%)	33 (8.0)	19 (7.1)	14 (9.5)	0.73 (0.35–1.50)	0.66 (0.31–1.40)
BARC 3b, n (%)	25 (6.0)	16 (6.0)	9 (6.1)	0.98 (0.42–2.27)	0.71 (0.28–1.78)
BARC 3c, n (%)	13 (3.1)	9 (3.4)	4 (2.7)	1.25 (0.38–4.12)	1.10 (0.32–3.79)
BARC 4, n (%)	0 (0.0)	0 (0.0)	0 (0.0)	N/A	N/A
BARC 5a, n (%)	5 (1.2)	5 (1.9)	0 (0.0)	N/A	N/A
BARC 5b, n (%)	2 (0.5)	1 (0.4)	1 (0.7)	0.55 (0.03–8.84)	0.49 (0.03–8.44)
BARC 2–5, n (%)	122 (29.5)	73 (27.3)	49 (33.3)	0.75 (0.49–1.16)	0.56 (0.34–0.94)

Abbreviations: BARC Bleeding Academic Research Consortium, TIMI Thrombolysis in Myocardial Infarction
*adjusted for age, sex, witnessed OHCA, low flow interval and extracorporeal CPR

Supplementary table S4. Comparison of ischemic outcomes between patients with out-of-hospital cardiac arrest treated with cangrelor and oral $P2Y_{12}$ inhibitors.

	OVERALL n=414	CANGRELOR n=267	ORAL $P2Y_{12}$ INHIBITOR n=147	Odds Ratio (95% CI)	Adjusted Odds Ratio (95% CI)*
Myocardial infarction, n (%)	0 (0.0)	0 (0.0)	0 (0.0)	N/A	N/A
Ischemic stroke, n (%)	14 (3.4)	10 (3.7)	4 (2.7)	1.39 (0.46–4.09)	1.44 (0.54–4.42)
Renal artery occlusion, n (%)	1 (0.2)	1 (0.4)	0 (0.0)	N/A	N/A
Peripheral artery occlusion, n (%)	9 (2.2)	1 (0.4)	8 (5.4)	0.07 (0.01–0.53)	0.04 (0.01–0.43)

*adjusted for age, sex, witnessed OHCA, low flow interval and extracorporeal CPR

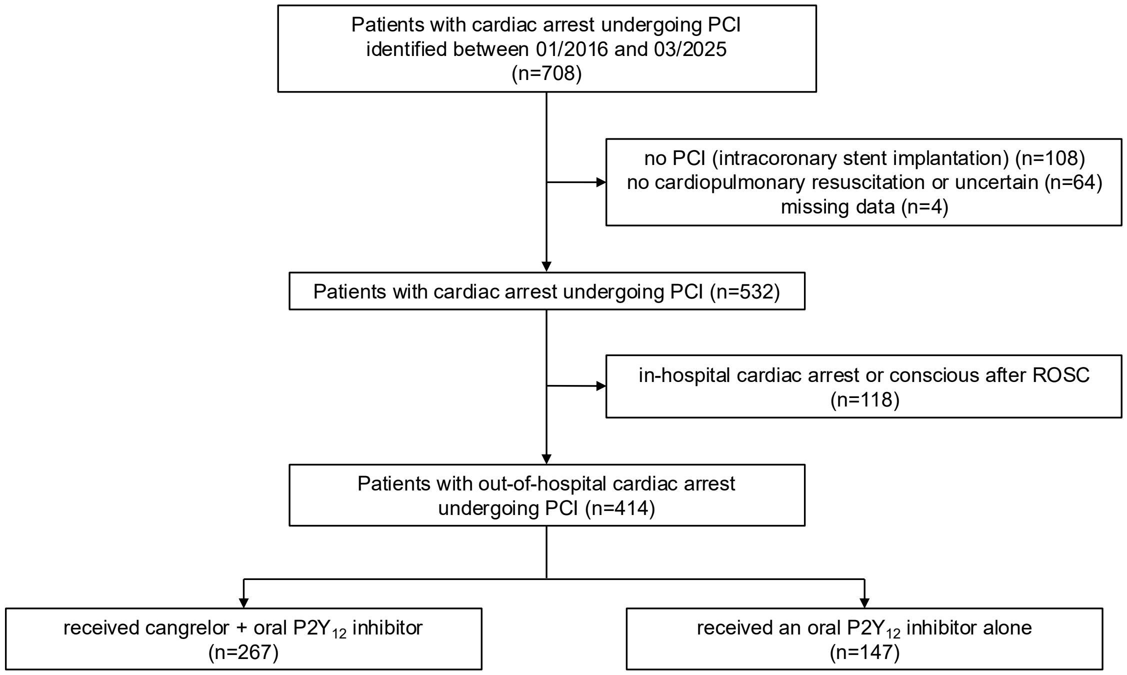

Supplementary figure S1. Study flowchart.

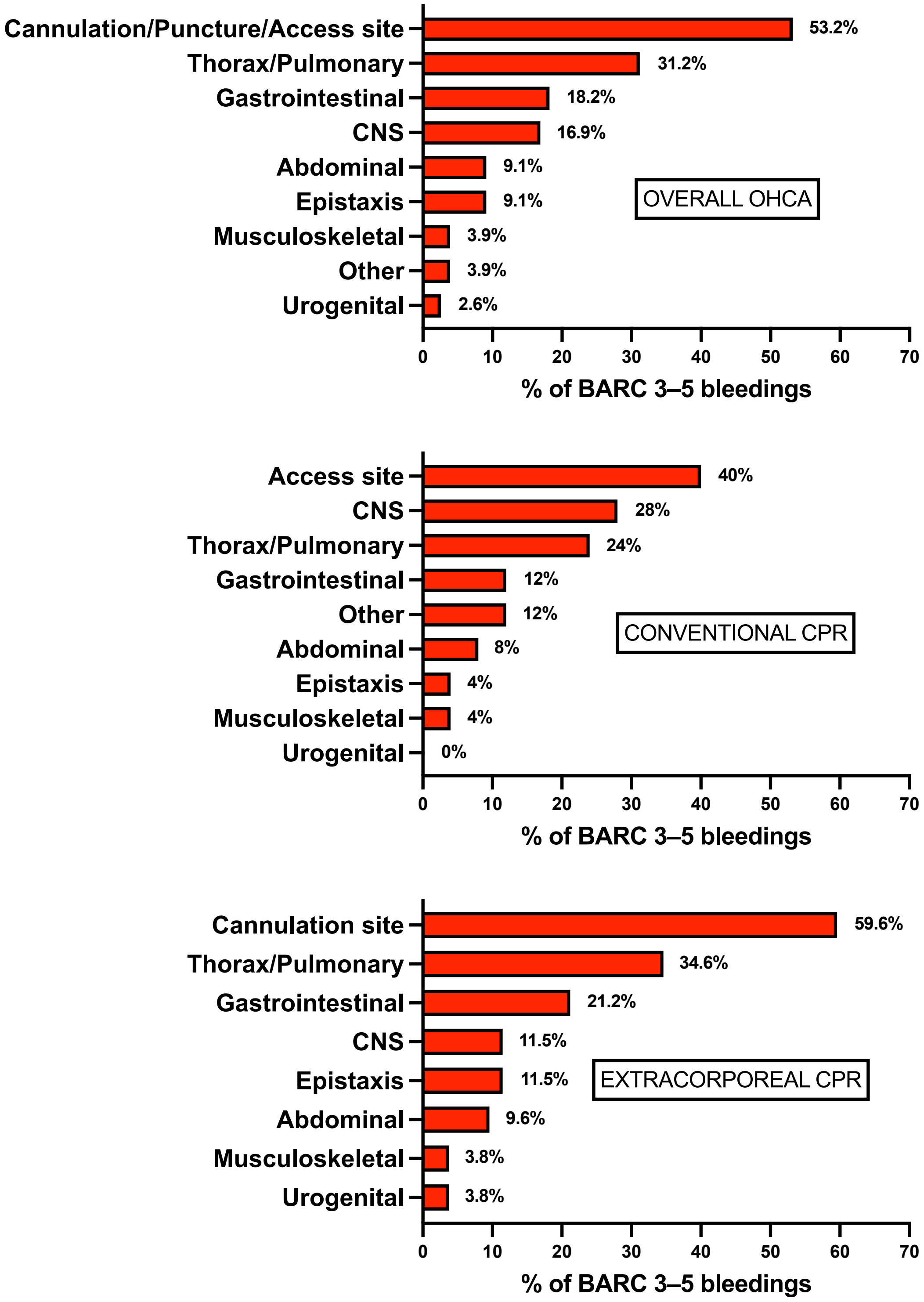

Supplementary figure S2. Bleeding sites of BARC 3–5 bleedings in the overall out-of-hospital cardiac arrest population, patients treated with conventional CPR and patients treated with extracorporeal CPR.

Numbers add up to more than 100% because some patients had more than one bleeding site.

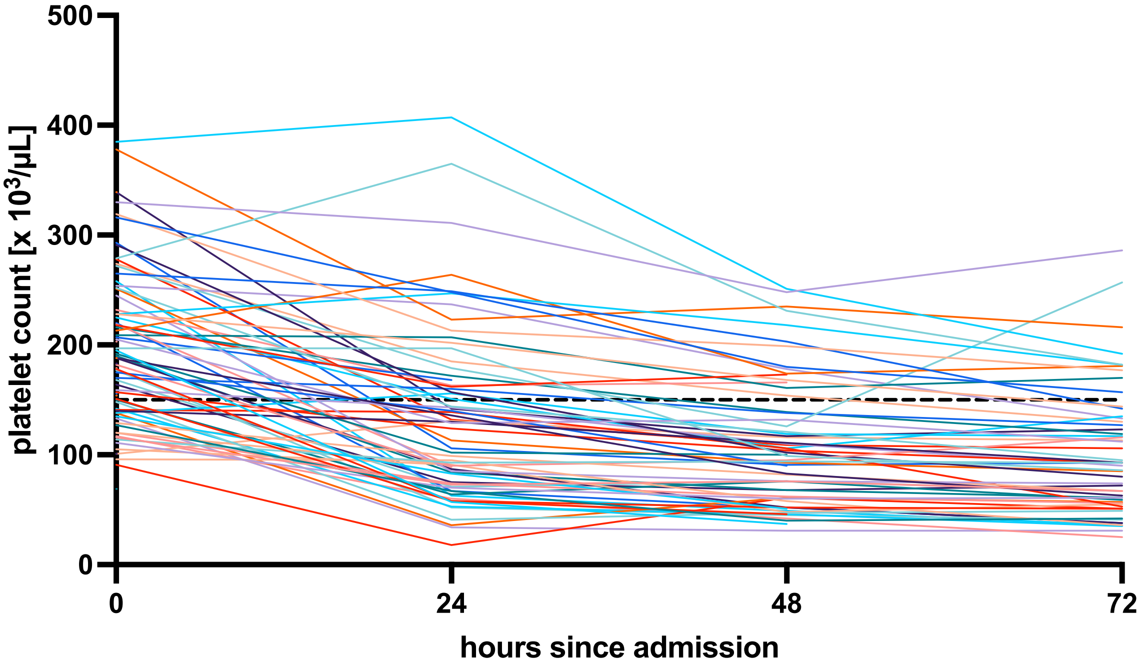

Supplementary figure S3. Platelet counts over the first 72 hours of admission in patients with a BARC 3–5 bleeding event.

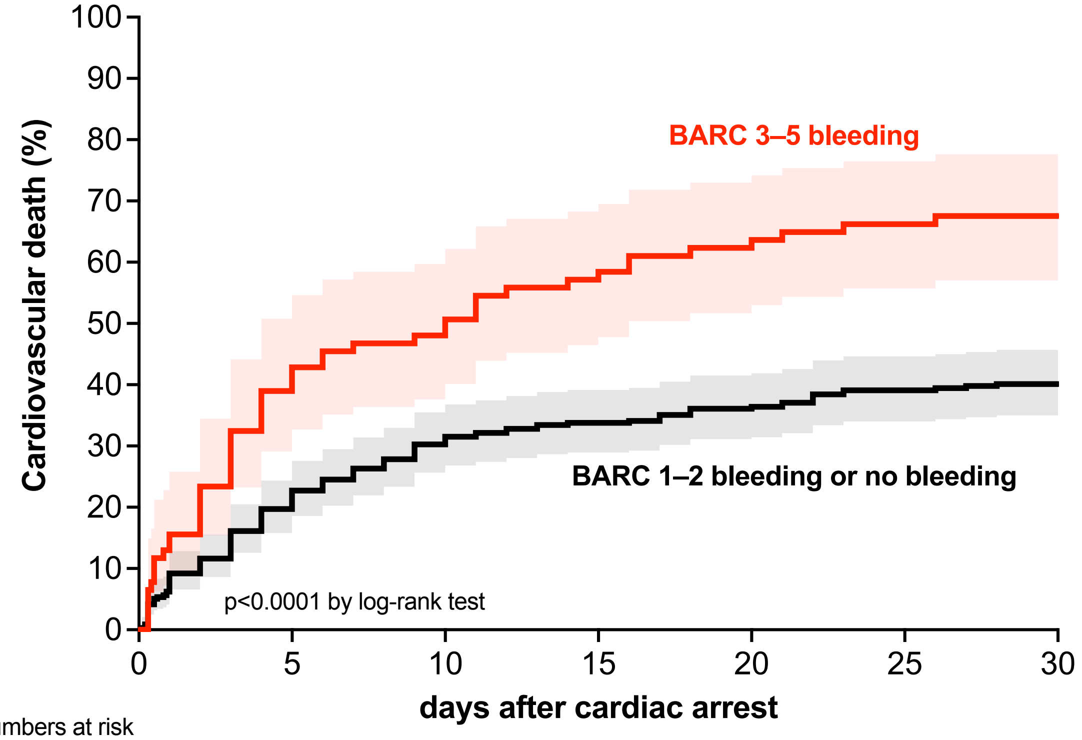

Numbers at risk							
BARC 1–2 or none	337	267	224	204	191	182	177
BARC 3–5	77	47	40	33	29	27	25

Supplementary figure S4. Kaplan-Meier estimates of the probability of cardiovascular death in patients with out-of-hospital cardiac arrest who had a BARC 1–2 or no bleeding event versus a BARC 3–5 bleeding event.

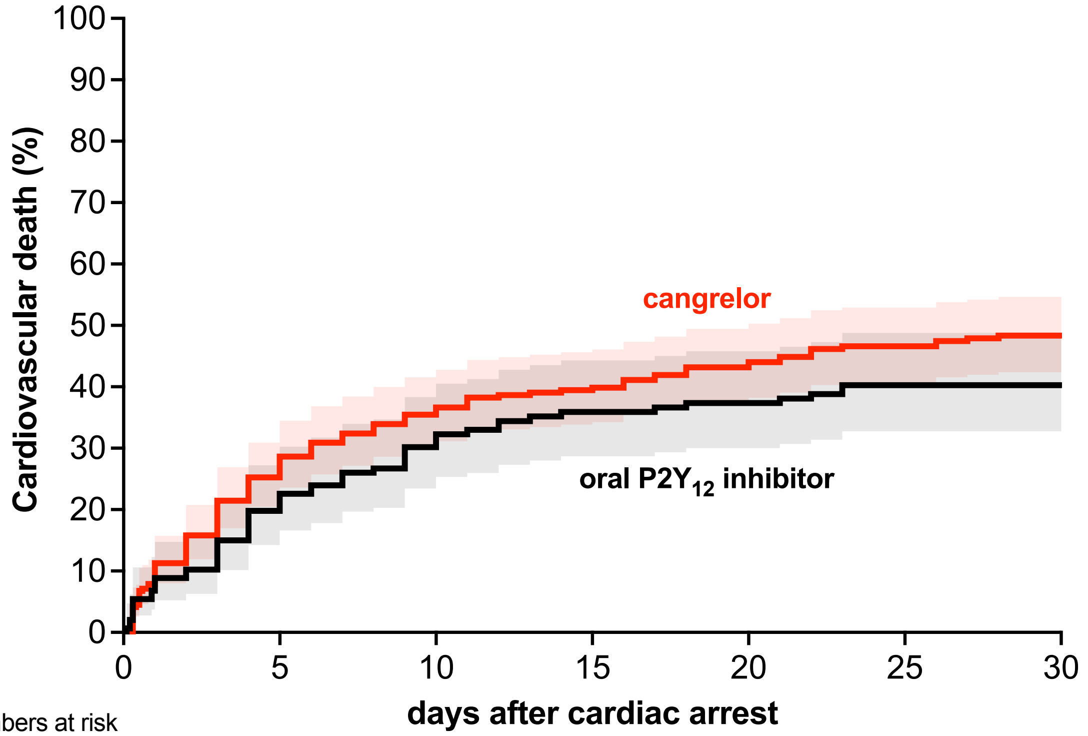

Numbers at risk

Cangrelor	267	198	164	148	134	125	120
oral $P2Y_{12}$i	147	116	100	89	87	84	82

Supplementary figure S5. Kaplan-Meier estimates of the probability of cardiovascular death in patients with out-of-hospital cardiac arrest treated with cangrelor and oral $P2Y_{12}$ inhibitors.
